# Associations Between Plasma Immunomodulatory and Inflammatory Mediators With VACS Index Scores Among Older HIV-Infected Adults on Antiretroviral Therapy

**DOI:** 10.3389/fimmu.2020.01321

**Published:** 2020-06-30

**Authors:** Thomas A. Premeaux, Shireen Javandel, Kalei R. J. Hosaka, Meredith Greene, Nicholas Therrien, Isabel E. Allen, Michael J. Corley, Victor G. Valcour, Lishomwa C. Ndhlovu

**Affiliations:** ^1^Department of Tropical Medicine, John A. Burns School of Medicine, University of Hawaii, Honolulu, HI, United States; ^2^Department of Neurology, Memory and Aging Center, University of California, San Francisco, San Francisco, CA, United States; ^3^Division of Geriatric Medicine, University of California, San Francisco, San Francisco, CA, United States; ^4^Department of Epidemiology and Biostatistics, University of California, San Francisco, San Francisco, CA, United States; ^5^Division of Infectious Diseases, Department of Medicine, Weill Cornell Medicine, New York, NY, United States

**Keywords:** HIV, aging, inflammation, morbidity, anti-retroviral therapy

## Abstract

The prevalence of age-related comorbidities is increased in people living with HIV, even in those well-controlled on combination antiretroviral therapy (ART). Persistent immune activation and inflammation may play pivotal roles in the pathogenesis; however, the burden of morbidities in the older HIV infected population may be exacerbated and driven by distinct mechanisms. In a cross sectional study of 45 HIV-infected participants 60 years or older, we examined the relationships between 14 immunomodulatory and inflammatory factors and the Veterans Aging Cohort Study (VACS) Index, a metric of multimorbidity and mortality comprised of age, CD4 count, hemoglobin, Fibrosis-4 [FIB-4], and estimated glomerular filtration rate [eGFR], by linear regression analysis. All participants were virally suppressed (<50 HIV RNA copies/mL), on ART, and primarily Caucasian (86.7%), and male (91.1%). Plasma levels of monocyte/macrophage-associated (neopterin, IP-10, sCD163, sCD14, and MCP-1) and glycan-binding immunomodulatory factors (galectin (Gal)-1, Gal-3, and Gal-9) were assessed, as well as inflammatory biomarkers previously linked to the VACS Index (i.e., CRP, cystatin C, TNF-α, TNFRI, IL-6, and D-dimer) for comparison. In regression analysis, higher VACS index scores were associated with higher levels of neopterin, cystatin C, TNFRI, and Gal-9 (all *p* < 0.05), potentially driven by correlations found with individual VACS components, including age, CD4 count, FIB-4, and eGFR. Gal-9, cystatin C, and TNFRI directly correlated with the extent of multimorbidity. Multiple correlations among markers were observed, suggesting an interplay of overlapping, but distinct, pathways. Collectively, in addition to cystatin C and TNFRI, both galectin-9 and neopterin, independently emerged as novel fluid markers of the VACS Index and burden of comorbidity and may further guide in understanding pathogenic mechanisms of age-related disorders in older HIV-infected individuals on suppressive ART.

## Introduction

In the current era of effective suppressive combination anti-retroviral therapy (ART) regimens people aging with HIV (PAWH) have an increased risk for and earlier onset of age-related comorbidities including cardiovascular, kidney, liver, bone, and neurologic disease (1, 2). Nearly half of the population living with HIV in the United States is older than 50 years of age and non-AIDS-defined events and age-related comorbidities are now the leading cause of mortality in the ART era (3–5). This demographic shift in the HIV-infected population further complicates the clinical care and management of PAWH, particularly as the increased burden of multimorbidity, frailty, geriatric syndromes, and polypharmacy become the norm (6, 7). Although multifactorial, evidence suggests chronic inflammation, particularly monocyte activation, is a key driver of early development of these comorbidities (8–11). This chronic inflammation is thought to stem from occult viral replication and the senescence, exhaustion, and premature aging of the immune system (12–14).

Soluble biomarkers of immune activation, inflammation, and coagulation, such as TNF-α and D-dimer, have been previously associated with clinical indices of morbidity and mortality in people with HIV on suppressive ART (15, 16). As organ system injury is strongly related to immune perturbations, linking clinical predictors and outcomes of morbidity and mortality with mediators of chronic inflammation and immune dysfunction can inform potential molecular pathways involved in the early onset of these comorbidities and, thus, guide therapeutic interventions. The Veterans Aging Cohort Study (VACS) Index is a validated predictor of morbidity and mortality for people with HIV by incorporating markers of organ system injury with traditional HIV disease estimates (17–19). The VACS Index is shown to correlate with age-related complications (i.e., frailty and cognitive impairment) and can inform mechanistic studies on the pathophysiologic effects of aging with HIV (15, 20–25). Evaluating soluble mediators with these clinical index scores and discovering novel correlations can provide a deeper understanding of the interplay between biological function and outcomes of morbidity and mortality in PAWH (26).

In order to inform our understanding of the biology of morbidity risk in older PAWH, we assessed correlations between the VACS Index and soluble monocyte/macrophage activation (neopterin, IP-10) and glycan-binding (galectins) immunomodulatory factors. Several of these factors are elevated in the plasma of untreated people living with HIV and, to a lesser degree, those on ART (27–29). Furthermore, many of these factors are shown to be involved in modulating metabolism and inflammation, directly involved in HIV replication, and linked to comorbidities in the general population (30–52). We investigated these immunomodulatory factors in addition to biomarkers traditionally associated with the VACS Index in a cohort of older adults living with HIV (over the age of 60) with long-term viral suppression to better understand the relationship between aging, HIV, and inflammation.

## Materials and Methods

### Participants

We conducted a cross sectional study of HIV-infected participants enrolled in the UCSF HIV Elders Study or HIV Over 60 Cohort. These studies were approved by the UCSF Institutional Review Board at the University of California, San Francisco. All participants were aged 60 or older, virally suppressed (defined as plasma HIV RNA <50 copies/mL), and reported adherence to ART for at least 12 months. All participants underwent neuropsychological testing and had endorsed cognitive symptoms on the Patient Assessment of Own Functioning questionnaire as previously described (53). Individuals with confounding conditions such as major neurological or psychiatric conditions were excluded. VACS index was calculated using age, current CD4 t-lymphocyte count, plasma HIV RNA level, hemoglobin, Fibrosis-4 (FIB-4) index, estimated glomerular filtration rate (eGFR), and active hepatitis C infection in a weighted manner, per the scoring system developed by the VACS Project Team (19). Self-reported current CD4 T-lymphocyte count was employed for two individuals as laboratory measures were not available. FIB-4 was calculated using routine liver function tests (AST and ALT), platelet count, and age. eGFR was calculated using serum creatinine levels, age, gender, and race.

### Quantification of Plasma Markers

Plasma aliquots were thawed and prepared following kit manufacturer guidelines. All samples were analyzed in duplicate. MCP-1, soluble (s)CD163, IP-10, galectin-1, galectin-3, galectin-9, IL-6, TNF-α, TNFR1, D-dimer, and cystatin C were measured using custom Luminex kits from (R&D Systems) and CRP was measured using the Procartaplex kit (Thermofisher). Data was acquired on a Luminex 200^TM^ analyzer (Luminex) and analyzed using MILLIPLEX^®^ Analyst software (Millipore). Neopterin and sCD14 were measured via ELISA (Neopterin competitive enzyme immunoassay, ALPCO, NH, USA; Human CD14 Quantikine ELISA kit, R&D Systems). Optical density was read with a microplate spectrophotometer (Bio-Rad) and data analysis, including four parameter logistic standard interpolation, was carried out using the online MyAssays Ltd. data analysis tool.

### Statistical Analysis

Demographic and HIV-related characteristics were described using the median, first quartile (Q1), and third quartile (Q3) for continuous variables, and frequency for categorical variables. Relationships among soluble markers and clinical parameters were examined by Pearson correlation for continuous variables or Spearman correlation for count variables. A multiple linear regression model was fit to examine predictors of the VACS index. Soluble marker values were log transformed to conform to normality prior to analysis. All statistical tests were performed with GraphPad Prism version 8.0 (Graphpad Software Inc., CA, USA) or SPSS version 26 (IBM SPSS Statistics, NY, USA). Correlation matrix was constructed using GraphPad Prism. Statistical significance is indicated as ^*^p < 0.05, ^**^p < 0.01, ^***^p < 0.001, ^****^p < 0.0001. *P*-values ≤ 0.100, but not significant, are noted as statistical trends.

## Results

Participants were 60 years of age or older, predominantly male (91.1%), and Caucasian (86.7%) (Table 1). All individuals were on ART and had undetectable plasma HIV RNA (viral load; VL). Most participants self-reported being infected with HIV for 20+ years (89%). More than half of participants (65%) had CD4 T-lymphocyte counts over 500 cells/μL; only one individual had CD4 T-lymphocyte count <200 cells/μL. Only one individual had a hemoglobin level <12 g/dL, three individuals had a FIB-4 score consistent with liver fibrosis (>3.25), 23% had some compromise in renal function (eGFR <60), and six had past co-infection with hepatitis C virus (HCV) and at least one active infection. We observed a mean VACS index score of 30 for the cohort. Based on prior reports, this suggests a 11.9% all-cause 5-years mortality risk (19, 54). Recent comorbid conditions of cohort participants were also assessed (Table 2), showing 14% were obese defined by a BMI >30, three had coronary disease, almost half (49%) had hyperlipidemia/hypercholesterolemia, more than half (58%) had hypertension, 22% had diabetes mellitus, one individual had liver disease, 22% had chronic obstructive pulmonary disease (COPD), three previously experienced kidney failure (two had stage III chronic kidney disease, and one had chronic renal insufficiency), and one individual had cancer.

**Table 1:** Demographics, HIV disease characteristics, and VACS components (*n* = 45).

**Variable**	
Age, yrs	65.0 (62.0, 66.0)
Gender (male)	41, 91.1%
Ethnicity (caucasian)	39, 86.7%
EDI, yrs	25.3 (23.0, 30)
HIV RNA <50 copies/mL	45, 100%
BMI^a^	26.5 (24.4, 28.4)
CD4 Nadir (cells/μL)^a^	173 (50, 243)
Current CD4 (cells/μL)^b^	623 (403, 804)
≥500	28, 65.1%
350–499	9, 20.9%
200–349	6, 13.0%
<200	1, 2.0%
Hemoglobin (g/dL)^b^	14.8 (14.2, 15.8)
FIB-4^a^	1.8 (1.2, 2.0)
<1.45	16, 36.4%
1.45–3.25	25, 56.8%
>3.25	3, 6.8%
eGFR^a^	73.7 (60.3, 87.2)
≥60	34, 77.3%
30–59.9	10, 22.7%
HCV infection	7, 15.6%
VACS index score^c^	30 (18, 39)

*Continuous variables are listed as mean (Q1, Q3) and categorical variables as n, %. EDI, estimated duration of infection; BMI, body mass index; FIB-4, fibrosis index 4; eGFR, estimated glomerular filtration rate; HCV, Hepatitis C virus*.

a *n = 36*,

b *n = 44*,

c *n = 43*.

**Table 2:** Prevalence of comorbid conditions.

**Comorbid condition**	**Recent**	**%**
Obesity (BMI > 30)^a^	6	13.6
Myocardial infarction / cardiac arrest	0	0
Heart failure	0	0
Coronary artery disease	3	6.7
Hyperlipidemia/hypercholesterolemia	22	48.9
Hypertension	24	53.3
Diabetes mellitus	9	20.0
Kidney failure	3	6.7
Liver disease^b^	1	2.3
Chronic obstructive pulmonary disease	10	22.2
Current smoker	6	13.3
Osteopenia or osteosclerosis	6	13.3
Cancer	1	2.2

*BMI, body mass index*;

a *n = 44*,

b *n = 43*.

We found several significant correlations between VACS Index scores and the plasma levels of inflammatory mediators, including the monocyte/macrophage-associated markers, glycan-binding immunomodulatory proteins, as well as inflammatory biomarkers previously linked to the VACS Index (Table 3). VACS index scores were significantly associated with neopterin (*r* = 0.40, *p* = 0.007), Gal-9 (*r* = 0.38, *p* = 0.012), cystatin C (*r* = 0.54, *p* = 0.0002), and TNFRI (*r* = 0.50, *p* = 0.0007). Furthermore, these correlations found with neopterin, Gal-9, cystatin C, and TNFRI remained statistically significant when adjusting for estimated duration of infection (EDI). Additionally, we observed trends between VACS index scores with IP-10 (*r* = 0.30, *p* = 0.051), Gal-1 (*r* = 0.30, *p* = 0.053), and TNF-α (*r* = 0.27, *p* = 0.082). However, markers previously being associated with the VACS index, including sCD163, sCD14, CRP, D-dimer, and IL-6, were not observed here.

**Table 3:** Soluble mediator correlations with VACS index scores.

**Parameter**	**VACS**	
	**Unadjusted**	**EDI adjusted**
	**Correlation coefficient (*****p*****-value)**	
Neopterin (nMol/L)	**0.40 (0.007)**	**0.43 (0.004)**
IP-10 (pg/mL)	0.30 (0.051)	0.29 (0.055)
sCD163 (pg/mL)	0.03 (0.840)	0.08 (0.637)
sCD14 (pg/mL)	−0.04 (0.781)	−0.07 (0.674)
MCP-1 (pg/mL)	0.18 (0.242)	0.15 (0.327)
Gal-1 (pg/mL)	0.30 (0.053)	0.28 (0.064)
Gal-3 (pg/mL)	0.18 (0.249)	0.20 (0.208)
Gal-9 (pg/mL)	**0.38 (0.012)**	**0.41 (0.006)**
D-dimer (pg/mL)	0.25 (0.101)	0.27 (0.075)
CRP (pg/mL)	−0.09 (0.587)	−0.09 (0.561)
Cystatin C (pg/mL)	**0.54 (0.0002)**	**0.53 (0.0003)**
IL-6 (pg/mL)	0.17 (0.291)	0.16 (0.312)
TNF-α (pg/mL)	0.27 (0.082)	0.27 (0.080)
TNFRI (pg/mL)	**0.50 (0.0007)**	**0.48 (0.002)**

*Soluble markers were log-transformed prior to Pearson correlation and multivariate analysis. EDI, estimated duration of infection. Significant values are bold*.

**Figure 1:**
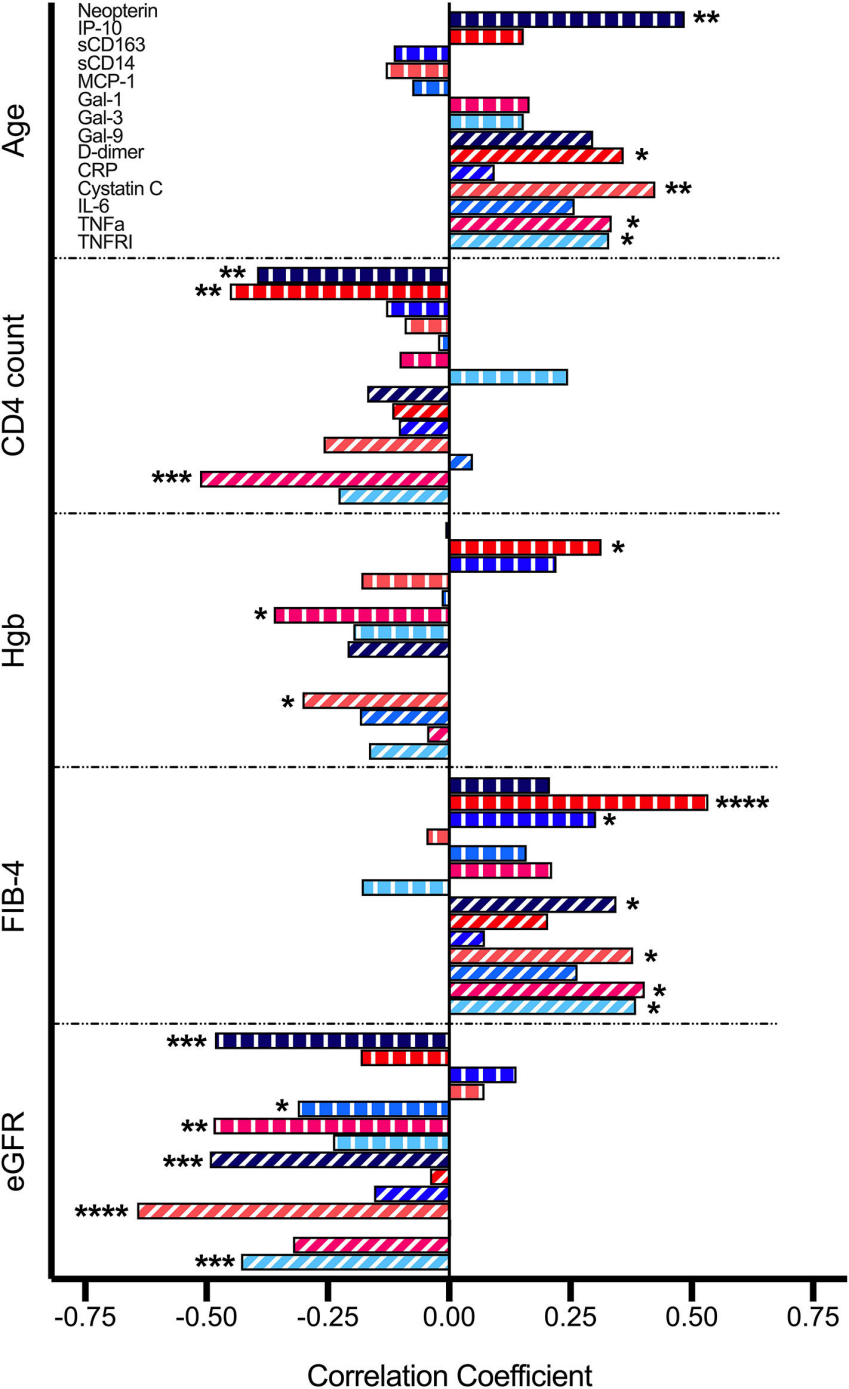
Correlations between soluble mediators and VACS Index components. Relationship between variables were analyzed by Pearson correlation. Statistical significance is indicated as ^*^p < 0.05, ^**^p < 0.01, ^***^p < 0.001, ^****^p < 0.0001.

**Figure 2:**
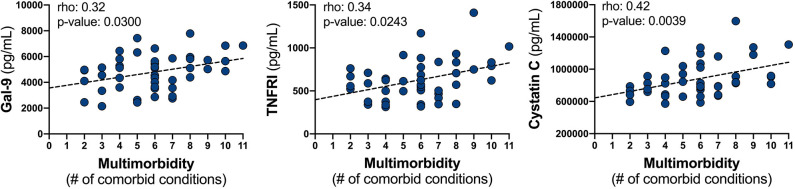
Relationship between soluble mediator levels and the burden of multimorbidity. Plasma levels of Gal-9, TNFRI, and cystatin C were correlated with the number of comorbid conditions by Spearman correlation; represented line is indicative only.

Upon correlation analysis of individual VACS components (Figure 1), we observed that levels of neopterin, cystatin C, TNF-α, and TNFRI were positively linearly associated with age (all *p* < 0.05). Neopterin, IP-10, and TNF-α inversely related with CD4 count (all *p* < 0.01). Hemoglobin levels were inversely associated with Gal-1 and cystatin C levels, but directly correlated with levels of IP-10 (all *p* < 0.05). Direct correlations between FIB-4 and IP-10, sCD163, Gal-9, cystatin C, TNF-α, and TNFRI were present (all *p* < 0.05). And finally, levels of neopterin, MCP-1, Gal-1, Gal-9, cystatin C, and TNFRI inversely related with eGFR (all *p* < 0.05). No soluble markers differed significantly among HCV status, except for higher Gal-3 levels observed in individuals with past HCV co-infection (*p* = 0.048; data not shown). Soluble markers levels were compared among individuals differing in CD4 nadir (>100, 100–200, >200) and no differences were observed (Supplementary Figure 1). Associations among sCD14, CRP, and IL-6 with individual VACS components were not found. We next evaluated these markers with the burden of multimorbidity (Figure 2). We observed that levels of Gal-9 (rho = 0.34, *p* = 0.021), TNFRI (rho = 0.36, *p* = 0.015), and cystatin C (rho = 0.44, *p* = 0.003) were directly correlated with the total number of comorbid conditions. Differences in soluble marker levels for specific comorbidities were also evaluated (Supplementary Figure 2). Only higher levels of CRP in individuals with obesity (*p* = 0.022) and higher Gal-3 with COPD (*p* = 0.023) were observed.

**Figure 3:**
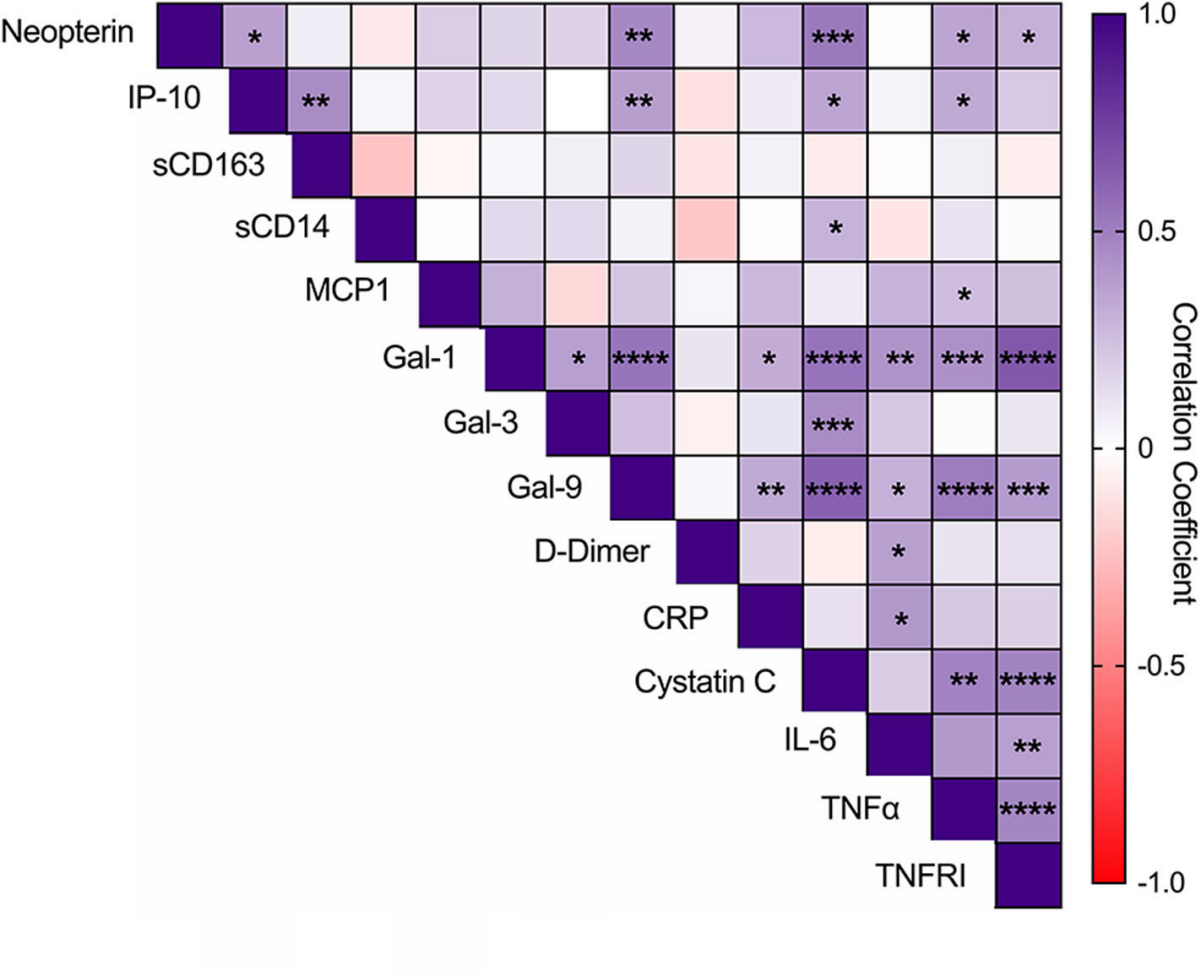
Intercorrelations among glycan-binding immunomodulators, myeloid-associated markers, and markers of coagulation and inflammation. Intercorrelations among soluble markers were analyzed by Pearson correlation. Statistical significance is indicated as ^*^p < 0.05, ^**^p < 0.01, ^***^p < 0.001, ^****^p < 0.0001.

We also conducted an exploratory analysis to determine if there exists an interplay of multiple immune pathways by evaluating intercorrelations among the 14 soluble mediators quantified (Figure 3). A positive interaction between neopterin and Gal-9 was noted (*p* < 0.001) and multiple correlations among several plasma markers were observed, including neopterin levels positively associating with IP-10, cystatin C, TNF-α, and TNFRI (*p* < 0.05). Gal-9 levels positively associated with IP-10, CRP, cystatin C, IL-6, TNF-α, TNFRI, and Gal-1 (*p* < 0.05). Among the other monocyte/macrophage activation markers, IP-10 also correlated with sCD163, cystatin C, and TNF-α; sCD14 only correlated with cystatin C (*p* < 0.05). And Gal-1 correlated with Gal-3, CRP, cystatin C, IL-6, TNF-α, and TNFRI (*p* < 0.05).

## Discussion

In an assessment of immunomodulatory factors in older PAWH on suppressive ART, we illuminated potential plasma mediators while highlighting the importance of previously evaluated markers that could lead to a better understanding of the biological complexity of the aging process within the context of HIV and assess those most vulnerable of experiencing morbidity and mortality. In addition to cystatin C and TNFRI, we identified a relationship between myeloid-associated and glycan-binding immunomodulators neopterin and Gal-9, and to a lesser extent IP-10 and Gal-1, with the VACS Index and several key VACS components. Furthermore, several of these factors correlated with the extent of comorbidities and with one another.

Chronic inflammation, particularly through monocyte/macrophage activation, is hypothesized as a key predictor of increased morbidity and all-cause mortality in HIV infection even in the context of viral suppression (55–57). Neopterin and IP-10 are markers of monocyte/macrophage activation that are associated with IFN-γ-induced pathways and demonstrate immune regulatory functions (58–61). Prior literature has demonstrated that both neopterin and IP-10 are implicated in the progression of HIV disease, principally associating with viremia, decreased CD4 counts, and all-cause mortality (62–64). We extend these observations by demonstrating that neopterin and, to a lesser extent, IP-10 associated with increased risk of mortality in older PAWH on suppressive ART. Furthermore, our correlative findings between eGFR, an indicator of kidney function, with these markers are consistent with previous findings demonstrating that neopterin associates with chronic kidney disease severity and IP-10 blockade promotes renal dysfunction (65, 66). Interventions that target monocyte activation, specifically IFN-γ-induced pathways, may potentially be useful in lowering chronic immune activation, and reduce morbidity incidence in PAWH during suppressive ART.

Glycan-glycan-binding protein mediated immune responses, particularly through the galectin family of proteins, is an emerging field in HIV research and implicated to play a role in HIV pathogenesis (67). Gal-9 and Gal-1, are demonstrated as drivers of HIV transcription and cell entry, respectively, as well as pro-inflammatory mediators (33–35, 37, 38). Gal-9 specifically has been shown to rapidly increase in the plasma as part of the cytokine storm in early HIV infection and remains elevated during chronic infection despite viral suppression (28). Furthermore, in the cerebrospinal fluid of people living with HIV higher Gal-9 levels were closely linked to increased viremia and immune activation as well as lower cognitive performance (68). Mechanistically, Gal-9 is hypothesized to potentially contribute to NK cell dysfunction and the non-specific activation of T cells in HIV infection (69, 70). Whether elevated endogenous Gal-9 levels contribute or sustain the state of chronic inflammation and immune activation and contribute to the progression of comorbid conditions during suppressive ART warrants further investigation. Here, we observed Gal-9 directly associated with morbidity risk and the extent of multimorbidity and our findings with VACS components are consistent with correlations previously demonstrated with FIB-4, a predictor of liver fibrosis, and eGFR in people without HIV (42, 44). As Gal-9 plays significant roles in both immune function and HIV transcription, elevated levels in PAWH may provide additional insights beyond biomarkers previously assessed with the VACS Index.

In this study we reveal additional inflammatory-induced pathways that could be contributing to the persistent inflammatory milieu and morbidity risk in treated HIV in the older population. We also conducted an evaluation of markers previously shown to associate with the VACS Index based on a diverse population in terms of age and ART treatment status. In our virally suppressed cohort, we found correlations among the VACS Index with cystatin C and TNFRI levels, as well as a trends with D-dimer and TNF-α, complementing the idea that perturbations in inflammation and coagulation mechanisms are linked to morbidity and mortality progression in PAWH (5). However, we did not observe VACS index correlations with CRP, IL-6, sCD14, and sCD163 as previously described (71). The absence of these correlations could stem from a low incidence of comorbid complications in our cohort that these biomarkers are found to be associated with, such as cardiovascular disease, bone disease, and cancer (72–78). Additionally, previous links between these biomarkers and the VACS Index were observed in relatively younger participants either with incomplete viral suppression (VL detectable but <500 c/mL) or an immunologic non-responder profile (20, 21).

As people living with HIV advance in age measures to determine increased risk for morbidity and mortality are warranted and soluble mediators may be particularly useful as screening tools to identify high-risk groups. However, the synergistic effects of HIV, aging, and multimorbidity complicates the ability to identify “universal” biomarkers. Previous studies demonstrate that PAWH display accelerated or early aging and accentuated inflammatory responses in advancing age, seroconversion at an earlier age associates with higher rates of multiple comorbidities, and specific mediators are associated with distinct comorbidities observed in HIV infection (79–82). Many comorbidities were common in our cohort, similar to other studies of older PAWH, which makes many of these soluble markers informative, as some are disease specific and others are common among many conditions. Furthermore, we discovered intercorrelations among many of the soluble mediators analyzed, suggesting an interplay of overlapping, but distinct, pathways, leading to the conclusion that a composite panel of clinical and immunomodulatory markers might be a more comprehensive approach for monitoring comorbid progression and the discovery of an effective means of intervention.

Chronic inflammation and immune activation are proposed as significant drivers of comorbidity development in PAWH, but this could be propelled by several factors, including biological aging, ARV regimens, HIV infection, or rather a synergistic effect. Chronic, low-grade inflammation occurs as people age, termed inflammaging, and soluble markers shown to be elevated with age, such as Gal-9 and neopterin, could be a reflection of this normal biological aging process (83). Adverse effects of several ARVs are linked to inflammation and the development of specific comorbidities; however, initiation of ART at diagnosis irrespective of CD4 count is now mandated given the overriding benefit of viral suppression in reducing transmission and lowering immune damage and higher states of inflammation (84–89). Current efforts to use ARV drugs that are safer and in combinations with anti-inflammatory agents are being pursued. To date, no therapy has been approved to decrease inflammation in PAWH despite numerous clinical trials targeting multiple proposed mechanisms of chronic inflammation in HIV (90–97). Any successes in interventions to lower immune activation and inflammation would be of significant value for PAWH and our panel of identified markers could potentially assist in monitoring interventions and identifying novel pathways to target.

Our study has several limitations that should be acknowledged. Our sample was relatively small and, thus, limited the power for multivariate regression analysis. Participants were primarily white males and, thus, we are limited in our ability to generalize our data to all affected populations. Our cohort consisted of participants who were virally suppressed and ≥ 60 years old, which may limit the predictive capabilities of the VACS Index. The availability of an age-matched comparison group comprised of people without HIV could have added more clarity to the relevance of these soluble mediators specifically in the context of aging with HIV. Additionally, many of these soluble mediators are shown to associate with age (98, 99). As calculations for VACS, FIB-4, and eGFR use age as a component adjustments for this variable in regression models were not feasible. Finally, we cannot determine the predictive nor diagnostic ability of these identified soluble mediators for specific non-AIDS clinical events.

In conclusion, we identified several key mediators in our population of PAWH on ART who were over 60, some of which were consistent with previous studies and in particular, novel factors such as neopterin and Gal-9. Further evaluation of these identified immunomodulatory factors could determine if suitable to implement in clinical practice to monitor those at an increased risk of developing comorbidities or mortality in aging HIV-infected individuals on suppressive ART. In light of our studies either independently or collectively, these immune markers could also emerge as novel mediators in understanding the pathogenic molecular mechanisms of age-related disorders in treated HIV that may ultimately lead to identifying potential targets to prevent, slow, or reverse these complications in this population.

## Data Availability Statement

The raw data supporting the conclusions of this article will be made available by the authors, without undue reservation.

## Ethics Statement

The studies involving human participants were reviewed and approved by UCSF Institutional Review Board at the University of California, San Francisco. The patients/participants provided their written informed consent to participate in this study.

## Author Contributions

TP contributed to biomarker data acquisition, statistical analysis, and wrote the manuscript. SJ, KH, IA, and MG contributed to clinical data acquisition and interpretation. NT contributed to biomarker data acquisition and interpretation. MC, VV, and LN contributed to study design and concept. All authors reviewed the manuscript.

## Conflict of Interest

VV has served as a consultant to ViiV Healthcare and Merck on issues related to HIV and aging. LN has served as an advisory board member to ViiV Healthcare related to ART and HIV. The remaining authors declare that the research was conducted in the absence of any commercial or financial relationships that could be construed as a potential conflict of interest.

## Supplementary Material

Supplementary Figure 1 Differences in soluble mediator levels in individuals according to CD4 nadir count. Associations among groups were analyzed via Kruskal-Wallis with Dunn's multiple comparison test. Neopterin concentration is in nMol/L.

Supplementary Figure 2 Differences in soluble mediator levels according to comorbidity. Neopterin concentration is in nMol/L. Associations among groups were analyzed via Mann-Whitney tests. Statistical significance is indicated as ^*^*p* < 0.05.

## References

[1] Deeks SG Tracy R Douek DC. Systemic effects of inflammation on health during chronic HIV infection. Immunity. (2013) 39:633–45. 10.1016/j.immuni.2013.10.001 24138880 PMC4012895

[2] Heaton RK Clifford DB Franklin DR Jr Woods SP Ake C Vaida F. HIV-associated neurocognitive disorders persist in the era of potent antiretroviral therapy: CHARTER study. Neurology. (2010) 75:2087–96. 10.1212/WNL.0b013e318200d727 21135382 PMC2995535

[3] Justice AC. HIV and aging: time for a new paradigm. Curr HIV/AIDS Rep. (2010) 7:69–76. 10.1007/s11904-010-0041-9 20425560

[4] Mills EJ Barnighausen T Negin J. HIV and aging–preparing for the challenges ahead. N Engl J Med. (2012) 366:1270–3. 10.1056/NEJMp1113643 22475591

[5] Montano M Bhasin S D'Aquila RT Erlandson KM Evans WJ Funderburg NT. Harvard HIV and aging workshop: perspectives and priorities from Claude D. Pepper Centers and Centers for AIDS Research. AIDS Res Hum Retroviruses. (2019) 35:999–1012. 10.1089/aid.2019.0130 31456412 PMC6862961

[6] Greene M Covinsky KE Valcour V Miao Y Madamba J Lampiris H. Geriatric syndromes in older HIV-infected adults. J Acquir Immune Defic Syndr. (2015) 69:161–7. 10.1097/QAI.0000000000000556 26009828 PMC4445476

[7] Guaraldi G Malagoli A Calcagno A Mussi C Celesia BM Carli F. The increasing burden and complexity of multi-morbidity and polypharmacy in geriatric HIV patients: a cross sectional study of people aged 65—74 years and more than 75 years. BMC Geriatr. (2018) 18:99. 10.1186/s12877-018-0789-0 29678160 PMC5910563

[8] Campbell JH Hearps AC Martin GE Williams KC Crowe SM. The importance of monocytes and macrophages in HIV pathogenesis, treatment, and cure. AIDS. (2014) 28:2175–87. 10.1097/QAD.0000000000000408 25144219 PMC6331181

[9] Hart BB Nordell AD Okulicz JF Palfreeman A Horban A Kedem E. Inflammation-related morbidity and mortality among HIV-positive adults: how extensive is it? J Acquir Immune Defic Syndr. (2018) 77:1–7. 10.1097/QAI.0000000000001554 28991883 PMC5720921

[10] Hunt PW Lee SA Siedner MJ. Immunologic biomarkers, morbidity, and mortality in treated HIV infection. J Infect Dis. (2016) 214(Suppl.2):S44–50. 10.1093/infdis/jiw275 27625430 PMC5021241

[11] Wilson EM Singh A Hullsiek KH Gibson D Henry WK Lichtenstein K. Monocyte-activation phenotypes are associated with biomarkers of inflammation and coagulation in chronic HIV infection. J Infect Dis. (2014) 210:1396–406. 10.1093/infdis/jiu275 24813472 PMC4207864

[12] Duffau P Wittkop L Lazaro E le Marec F Cognet C Blanco P. Association of immune-activation and senescence markers with non-AIDS-defining comorbidities in HIV-suppressed patients. AIDS. (2015) 29:2099–108. 10.1097/QAD.0000000000000807 26544576

[13] Desai S Landay A. Early immune senescence in HIV disease. Curr HIV/AIDS Rep. (2010) 7:4–10. 10.1007/s11904-009-0038-4 20425052 PMC3739442

[14] Appay V Kelleher AD. Immune activation and immune aging in HIV infection. Curr Opin HIV AIDS. (2016) 11:242–9. 10.1097/COH.0000000000000240 26845675

[15] Mooney S Tracy R Osler T Grace C. Elevated biomarkers of inflammation and coagulation in patients with HIV are associated with higher framingham and VACS risk index scores. PLoS ONE. (2015) 10:e0144312. 10.1371/journal.pone.0144312 26641655 PMC4671539

[16] Duffau P Ozanne A Bonnet F Lazaro E Cazanave C Blanco P. Multimorbidity, age-related comorbidities and mortality: association of activation, senescence and inflammation markers in HIV adults. AIDS. (2018) 32:1651–60. 10.1097/QAD.0000000000001875 29762168

[17] Tate JP Justice AC Hughes MD Bonnet F Reiss P Mocroft A. An internationally generalizable risk index for mortality after 1 year of antiretroviral therapy. AIDS. (2013) 27:563–72. 10.1097/QAD.0b013e32835b8c7f 23095314 PMC4283204

[18] Bebu I Tate J Rimland D Mesner O Macalino GE Ganesan A. The VACS index predicts mortality in a young, healthy HIV population starting highly active antiretroviral therapy. J Acquir Immune Defic Syndr. (2014) 65:226–30. 10.1097/QAI.0000000000000045 24226058 PMC4091811

[19] Justice AC Modur SP Tate JP Althoff KN Jacobson LP Gebo KA. Predictive accuracy of the Veterans Aging Cohort Study index for mortality with HIV infection: a North American cross cohort analysis. J Acquir Immune Defic Syndr. (2013) 62:149–63. 10.1097/QAI.0b013e31827df36c 23187941 PMC3619393

[20] Williams B Livak B Bahk M Keating SM Adeyemi OM. Short communication: SCD14 and SCD163 levels are correlated with VACS index scores: initial data from the Blunted Immune Recovery in CORE Patients with HIV (BIRCH) Cohort. AIDS Res Hum Retroviruses. (2016) 32:144–7. 10.1089/aid.2015.0012 26366931

[21] Justice AC Freiberg MS Tracy R Kuller L Tate JP Goetz MB. Does an index composed of clinical data reflect effects of inflammation, coagulation, and monocyte activation on mortality among those aging with HIV? Clin Infect Dis. (2012) 54:984–94. 10.1093/cid/cir989 22337823 PMC3297653

[22] Marquine MJ Montoya JL Umlauf A Fazeli PL Gouaux B Heaton RK. The Veterans Aging Cohort Study (VACS) index and neurocognitive change: a longitudinal study. Clin Infect Dis. (2016) 63:694–702. 10.1093/cid/ciw328 27199461 PMC4981756

[23] Akgun KM Tate JP Crothers K Crystal S Leaf DA Womack J. An adapted frailty-related phenotype and the VACS index as predictors of hospitalization and mortality in HIV-infected and uninfected individuals. J Acquir Immune Defic Syndr. (2014) 67:397–404. 10.1097/QAI.0000000000000341 25202921 PMC4213242

[24] Escota GV Patel P Brooks JT Bush T Conley L Baker J. Short communication: the Veterans Aging Cohort Study Index is an effective tool to assess baseline frailty status in a contemporary cohort of HIV-infected persons. AIDS Res Hum Retroviruses. (2015) 31:313–7. 10.1089/aid.2014.0225 25495766

[25] Marquine MJ Sakamoto M Dufour C Rooney A Fazeli P Umlauf A. The impact of ethnicity/race on the association between the Veterans Aging Cohort Study (VACS) Index and neurocognitive function among HIV-infected persons. J Neurovirol. (2016) 22:442–54. 10.1007/s13365-015-0411-6 26679535 PMC4912471

[26] Tenorio AR Zheng Y Bosch RJ Krishnan S Rodriguez B Hunt PW Soluble markers of inflammation and coagulation but not T-cell activation predict non-AIDS-defining morbid events during suppressive antiretroviral treatment. J Infect Dis. (2014) 210:1248–59. 10.1093/infdis/jiu254 24795473 PMC4192039

[27] Wirleitner B Schroecksnadel K Winkler C Fuchs D. Neopterin in HIV-1 infection. Mol Immunol. (2005) 42:183–94. 10.1016/j.molimm.2004.06.017 15488607

[28] Tandon R Chew GM Byron MM Borrow P Niki T Hirashima M. Galectin-9 is rapidly released during acute HIV-1 infection and remains sustained at high levels despite viral suppression even in elite controllers. AIDS Res Hum Retroviruses. (2014) 30:654–64. 10.1089/aid.2014.0004 24786365 PMC4077009

[29] Pastor L Casellas A Ruperez M Carrillo J Maculuve S Jairoce C. Interferon-gamma-Inducible Protein 10 (IP-10) as a screening tool to optimize human immunodeficiency virus RNA monitoring in resource-limited settings. Clin Infect Dis. (2017) 65:1670–5. 10.1093/cid/cix600 29020145 PMC5850521

[30] Brinchmann MF Patel DM Iversen MH. The role of galectins as modulators of metabolism and inflammation. Mediators Inflamm. (2018) 2018:9186940. 10.1155/2018/9186940 29950926 PMC5987346

[31] Liu M Guo S Hibbert JM Jain V Singh N Wilson NO. CXCL10/IP-10 in infectious diseases pathogenesis and potential therapeutic implications. Cytokine Growth Factor Rev. (2011) 22:121–30. 10.1016/j.cytogfr.2011.06.001 21802343 PMC3203691

[32] Gieseg SP Baxter-Parker G Lindsay A. Neopterin, inflammation, and oxidative stress: what could we be missing? Antioxidants. (2018) 7:80. 10.3390/antiox7070080 29949851 PMC6071275

[33] Abdel-Mohsen M Chavez L Tandon R Chew GM Deng X Danesh A. Human Galectin-9 Is a potent mediator of HIV transcription and reactivation. PLoS Pathog. (2016) 12:e1005677. 10.1371/journal.ppat.1005677 27253379 PMC4890776

[34] Bi S Hong PW Lee B Baum LG. Galectin-9 binding to cell surface protein disulfide isomerase regulates the redox environment to enhance T-cell migration and HIV entry. Proc Natl Acad Sci USA. (2011) 108:10650–5. 10.1073/pnas.1017954108 21670307 PMC3127870

[35] Mercier S St-Pierre C Pelletier I Ouellet M Tremblay MJ Sato S. Galectin-1 promotes HIV-1 infectivity in macrophages through stabilization of viral adsorption. Virology. (2008) 371:121–9. 10.1016/j.virol.2007.09.034 18028978

[36] Okamoto M Hidaka A Toyama M Baba M. Galectin-3 is involved in HIV-1 expression through NF-kappaB activation and associated with Tat in latently infected cells. Virus Res. (2019) 260:86–93. 10.1016/j.virusres.2018.11.012 30481548

[37] Ouellet M Mercier S Pelletier I Bounou S Roy J Hirabayashi J. Galectin-1 acts as a soluble host factor that promotes HIV-1 infectivity through stabilization of virus attachment to host cells. J Immunol. (2005) 174:4120–6. 10.4049/jimmunol.174.7.4120 15778371

[38] St-Pierre C Manya H Ouellet M Clark GF Endo T Tremblay MJ. Host-soluble galectin-1 promotes HIV-1 replication through a direct interaction with glycans of viral gp120 and host CD4. J Virol. (2011) 85:11742–51. 10.1128/JVI.05351-11 21880749 PMC3209312

[39] Wang SF Tsao CH Lin YT Hsu DK Chiang ML Lo CH. Galectin-3 promotes HIV-1 budding via association with Alix and Gag p6. Glycobiology. (2014) 24:1022–35. 10.1093/glycob/cwu064 24996823 PMC4181451

[40] Avanzas P Arroyo-Espliguero R Kaski JC. Neopterin and cardiovascular disease: growing evidence for a role in patient risk stratification. Clin Chem. (2009) 55:1056–7. 10.1373/clinchem.2009.127084 19395434

[41] Drechsler C Delgado G Wanner C Blouin K Pilz S Tomaschitz A. Galectin-3, renal function, and clinical outcomes: results from the LURIC and 4D studies. J Am Soc Nephrol. (2015) 26:2213–21. 10.1681/ASN.2014010093 25568176 PMC4552104

[42] Fujita K Niki T Nomura T Oura K Tadokoro T Sakamoto T. Correlation between serum galectin-9 levels and liver fibrosis. J Gastroenterol Hepatol. (2018) 33:492–9. 10.1111/jgh.13851 28618039

[43] Gudowska M Gruszewska E Cylwik B Panasiuk A Rogalska M Flisiak R. Galectin-3 concentration in liver diseases. Ann Clin Lab Sci. (2015) 45:669–73. 26663797

[44] Kurose Y Wada J Kanzaki M Teshigawara S Nakatsuka A Murakami K. Serum galectin-9 levels are elevated in the patients with type 2 diabetes and chronic kidney disease. BMC Nephrol. (2013) 14:23. 10.1186/1471-2369-14-23 23339460 PMC3556305

[45] Rebholz CM Selvin E Liang M Ballantyne CM Hoogeveen RC Aguilar D. Plasma galectin-3 levels are associated with the risk of incident chronic kidney disease. Kidney Int. (2018) 93:252–9. 10.1016/j.kint.2017.06.028 28865675 PMC5750096

[46] Seropian IM Gonzalez GE Maller SM Berrocal DH Abbate A Rabinovich GA. Galectin-1 as an emerging mediator of cardiovascular inflammation: mechanisms and therapeutic opportunities. Mediators Inflamm. (2018) 2018:8696543. 10.1155/2018/8696543 30524200 PMC6247465

[47] Unuvar S Aslanhan H. Clinical significance of increased serum neopterin in chronic kidney failure as a biomarker of cell-mediated immunity. J Med Biochem. (2019) 38:1–5. 10.2478/jomb-2018-0019 30820177 PMC6298458

[48] Vaidya VS Ferguson MA Bonventre JV. Biomarkers of acute kidney injury. Annu Rev Pharmacol Toxicol. (2008) 48:463–93. 10.1146/annurev.pharmtox.48.113006.094615 17937594 PMC2742480

[49] van den Borne P Quax PH Hoefer IE Pasterkamp G. The multifaceted functions of CXCL10 in cardiovascular disease. Biomed Res Int. (2014) 2014:893106. 10.1155/2014/893106 24868552 PMC4017714

[50] Wilmer A Nolchen B Tilg H Herold M Pechlaner C Judmaier G. Serum neopterin concentrations in chronic liver disease. Gut. (1995) 37:108–12. 10.1136/gut.37.1.108 7672657 PMC1382779

[51] You CR Park SH Jeong SW Woo HY Bae SH Choi JY. Serum IP-10 levels correlate with the severity of liver histopathology in patients infected with genotype-1 HCV. Gut Liver. (2011) 5:506–12. 10.5009/gnl.2011.5.4.506 22195251 PMC3240796

[52] Zhong X Qian X Chen G Song X. The role of galectin-3 in heart failure and cardiovascular disease. Clin Exp Pharmacol Physiol. (2019) 46:197–203. 10.1111/1440-1681.13048 30372548

[53] Chang K Premeaux TA Cobigo Y Milanini B Hellmuth J Rubin LH. Plasma inflammatory biomarkers link to diffusion tensor imaging metrics in virally suppressed HIV-infected individuals. AIDS. (2020) 34:203–13. 10.1097/QAD.0000000000002404 31634200 PMC6933087

[54] Brown ST Tate JP Kyriakides TC Kirkwood KA Holodniy M Goulet JL. The VACS index accurately predicts mortality and treatment response among multi-drug resistant HIV infected patients participating in the options in management with antiretrovirals (OPTIMA) study. PLoS ONE. (2014) 9:e92606. 10.1371/journal.pone.0092606 24667813 PMC3965438

[55] Knudsen TB Ertner G Petersen J Moller HJ Moestrup SK Eugen-Olsen J. Plasma soluble CD163 level independently predicts all-cause mortality in HIV-1-infected individuals. J Infect Dis. (2016) 214:1198–204. 10.1093/infdis/jiw263 27354366

[56] High KP Brennan-Ing M Clifford DB Cohen MH Currier J Deeks SG. HIV and aging: state of knowledge and areas of critical need for research. A report to the NIH Office of AIDS Research by the HIV and Aging Working Group. J Acquir Immune Defic Syndr. (2012) 60(Suppl.1):S1–18. 10.1097/QAI.0b013e31825a3668 22688010 PMC3413877

[57] Sandler NG Wand H Roque A Law M Nason MC Nixon DE. Plasma levels of soluble CD14 independently predict mortality in HIV infection. J Infect Dis. (2011) 203:780–90. 10.1093/infdis/jiq118 21252259 PMC3071127

[58] Ramirez LA Arango TA Thompson E Naji M Tebas P Boyer JD. High IP-10 levels decrease T cell function in HIV-1-infected individuals on ART. J Leukoc Biol. (2014) 96:1055–63. 10.1189/jlb.3A0414-232RR 25157027 PMC4226794

[59] Huber C Batchelor JR Fuchs D Hausen A Lang A Niederwieser D. Immune response-associated production of neopterin. Release from macrophages primarily under control of interferon-gamma. J Exp Med. (1984) 160:310–6. 10.1084/jem.160.1.310 6429267 PMC2187425

[60] Hoffmann G Frede S Kenn S Smolny M Wachter H Fuchs D. Neopterin-induced tumor necrosis factor-alpha synthesis in vascular smooth muscle cells *in vitro*. Int Arch Allergy Immunol. (1998) 116:240–5. 10.1159/000023950 9693272

[61] Hoffmann G Kenn S Wirleitner B Deetjen C Frede S Smolny M. Neopterin induces nitric oxide-dependent apoptosis in rat vascular smooth muscle cells. Immunobiology. (1998) 199:63–73. 10.1016/S0171-2985(98)80064-8 9717668

[62] Lei J Yin X Shang H Jiang Y. IP-10 is highly involved in HIV infection. Cytokine. (2019) 115:97–103. 10.1016/j.cyto.2018.11.018 30472104

[63] Mhandire K Mlambo T Zijenah LS Duri K Mateveke K Tshabalala M. Plasma IP-10 concentrations correlate positively with viraemia and inversely with CD4 counts in untreated HIV infection. Open AIDS J. (2017) 11:24–31. 10.2174/1874613601711010024 28553429 PMC5427702

[64] Valverde-Villegas JM de Medeiros RM Ellwanger JH Santos BR Melo MG Almeida SEM. High CXCL10/IP-10 levels are a hallmark in the clinical evolution of the HIV infection. Infect Genet Evol. (2018) 57:51–8. 10.1016/j.meegid.2017.11.002 29122683

[65] Yadav AK Sharma V Jha V. Association between serum neopterin and inflammatory activation in chronic kidney disease. Mediators Inflamm. (2012) 2012:476979. 10.1155/2012/476979 22969169 PMC3433148

[66] Nakaya I Wada T Furuichi K Sakai N Kitagawa K Yokoyama H. Blockade of IP-10/CXCR3 promotes progressive renal fibrosis. Nephron Exp Nephrol. (2007) 107:e12–21. 10.1159/000106505 17671396

[67] Colomb F Giron LB Trbojevic-Akmacic I Lauc G Abdel-Mohsen M. Breaking the Glyco-Code of HIV persistence and immunopathogenesis. Curr HIV/AIDS Rep. (2019) 16:151–68. 10.1007/s11904-019-00433-w 30707400 PMC6441623

[68] Premeaux TA D'Antoni ML Abdel-Mohsen M Pillai SK Kallianpur KJ Nakamoto BK. Elevated cerebrospinal fluid Galectin-9 is associated with central nervous system immune activation and poor cognitive performance in older HIV-infected individuals. J Neurovirol. (2019) 25:150–61. 10.1007/s13365-018-0696-3 30478799 PMC6506351

[69] Colomb F Giron LB Premeaux TA Mitchell BI Niki T Papasavvas E. Galectin-9 Mediates HIV Transcription by Inducing TCR-Dependent ERK Signaling. Front Immunol. (2019) 10:267. 10.3389/fimmu.2019.00267 30842775 PMC6391929

[70] Jost S Moreno-Nieves UY Garcia-Beltran WF Rands K Reardon J Toth I. Dysregulated Tim-3 expression on natural killer cells is associated with increased Galectin-9 levels in HIV-1 infection. Retrovirology. (2013) 10:74. 10.1186/1742-4690-10-74 23866914 PMC3750478

[71] Miller CJ Baker JV Bormann AM Erlandson KM Huppler Hullsiek K Justice AC. Adjudicated morbidity and mortality outcomes by age among individuals with HIV infection on suppressive antiretroviral therapy. PLoS ONE. (2014) 9:e95061. 10.1371/journal.pone.0095061 24728071 PMC3984283

[72] Borges AH O'Connor JL Phillips AN Ronsholt FF Pett S Vjecha MJ. Determinants of IL-6 levels during HIV infection. J Int AIDS Soc. (2014) 17(4 Suppl.3):19482. 10.7448/IAS.17.4.19482 25393991 PMC4224801

[73] Borges AH Silverberg MJ Wentworth D Grulich AE Fatkenheuer G Mitsuyasu R. Predicting risk of cancer during HIV infection: the role of inflammatory and coagulation biomarkers. AIDS. (2013) 27:1433–41. 10.1097/QAD.0b013e32835f6b0c 23945504 PMC4046103

[74] Duprez DA Neuhaus J Kuller LH Tracy R Belloso W De Wit S. Inflammation, coagulation and cardiovascular disease in HIV-infected individuals. PLoS ONE. (2012) 7:e44454. 10.1371/journal.pone.0044454 22970224 PMC3438173

[75] Ford ES Greenwald JH Richterman AG Rupert A Dutcher L Badralmaa Y. Traditional risk factors and D-dimer predict incident cardiovascular disease events in chronic HIV infection. AIDS. (2010) 24:1509–17. 10.1097/QAD.0b013e32833ad914 20505494 PMC2884071

[76] Hileman CO Labbato DE Storer NJ Tangpricha V McComsey GA. Is bone loss linked to chronic inflammation in antiretroviral-naive HIV-infected adults? A 48-week matched cohort study. AIDS. (2014) 28:1759–67. 10.1097/QAD.0000000000000320 24871454 PMC4404700

[77] Musselwhite LW Sheikh V Norton TD Rupert A Porter BO Penzak SR. Markers of endothelial dysfunction, coagulation and tissue fibrosis independently predict venous thromboembolism in HIV. AIDS. (2011) 25:787–95. 10.1097/QAD.0b013e3283453fcb 21412059 PMC4681576

[78] Triant VA Lee H Hadigan C Grinspoon SK. Increased acute myocardial infarction rates and cardiovascular risk factors among patients with human immunodeficiency virus disease. J Clin Endocrinol Metab. (2007) 92:2506–12. 10.1210/jc.2006-2190 17456578 PMC2763385

[79] Gallant J Hsue PY Shreay S Meyer N. Comorbidities among US patients with prevalent HIV infection-A trend analysis. J Infect Dis. (2017) 216:1525–33. 10.1093/infdis/jix518 29253205

[80] Guaraldi G Zona S Brothers TD Carli F Stentarelli C Dolci G. Aging with HIV vs. HIV seroconversion at older age: a diverse population with distinct comorbidity profiles. PLoS ONE. (2015) 10:e0118531. 10.1371/journal.pone.0118531 25874806 PMC4395353

[81] Schouten J Wit FW Stolte IG Kootstra NA van der Valk M Geerlings SE. Cross-sectional comparison of the prevalence of age-associated comorbidities and their risk factors between HIV-infected and uninfected individuals: the AGEhIV cohort study. Clin Infect Dis. (2014) 59:1787–97. 10.1093/cid/ciu701 25182245

[82] Hasse B Ledergerber B Furrer H Battegay M Hirschel B Cavassini M Morbidity and aging in HIV-infected persons: the Swiss HIV cohort study. Clin Infect Dis. (2011) 53:1130–9. 10.1093/cid/cir626 21998280

[83] Franceschi C Garagnani P Parini P Giuliani C Santoro A. Inflammaging: a new immune-metabolic viewpoint for age-related diseases. Nat Rev Endocrinol. (2018) 14:576–90. 10.1038/s41574-018-0059-4 30046148

[84] Brown TT Qaqish RB. Antiretroviral therapy and the prevalence of osteopenia and osteoporosis: a meta-analytic review. AIDS. (2006) 20:2165–74. 10.1097/QAD.0b013e32801022eb 17086056

[85] Daugas E Rougier JP Hill G. HAART-related nephropathies in HIV-infected patients. Kidney Int. (2005) 67:393–403. 10.1111/j.1523-1755.2005.67096.x 15673287

[86] Friis-Moller N Sabin CA Weber R d'Arminio Monforte A El-Sadr WM Reiss P. Combination antiretroviral therapy and the risk of myocardial infarction. N Engl J Med. (2003) 349:1993–2003. 10.1056/NEJMoa030218 14627784

[87] Villarroya F Domingo P Giralt M. Drug-induced lipotoxicity: lipodystrophy associated with HIV-1 infection and antiretroviral treatment. Biochim Biophys Acta. (2010) 1801:392–9. 10.1016/j.bbalip.2009.09.018 19800025

[88] Group ISS Lundgren JD Babiker AG Gordin F Emery S Grund B Initiation of Antiretroviral Therapy in Early Asymptomatic HIV Infection. N Engl J Med. (2015) 373:795–807. 10.1056/NEJMoa1506816 26192873 PMC4569751

[89] Lifson AR Grund B Gardner EM Kaplan R Denning E Engen N. Improved quality of life with immediate versus deferred initiation of antiretroviral therapy in early asymptomatic HIV infection. AIDS. (2017) 31:953–63. 10.1097/QAD.0000000000001417 28121710 PMC5373969

[90] group As. Vorapaxar for HIV-associated inflammation and coagulopathy (ADVICE): a randomised, double-blind, placebo-controlled trial. Lancet HIV. (2018) 5:e553–9. 10.1016/S2352-3018(18)30214-5 30257802 PMC6237199

[91] Aberg JA Sponseller CA Ward DJ Kryzhanovski VA Campbell SE Thompson MA. Pitavastatin versus pravastatin in adults with HIV-1 infection and dyslipidaemia (INTREPID): 12 week and 52 week results of a phase 4, multicentre, randomised, double-blind, superiority trial. Lancet HIV. (2017) 4:e284–94. 10.1016/S2352-3018(17)30075-9 28416195

[92] Toribio M Fitch KV Sanchez L Burdo TH Williams KC Sponseller CA. Effects of pitavastatin and pravastatin on markers of immune activation and arterial inflammation in HIV. AIDS. (2017) 31:797–806. 10.1097/QAD.0000000000001427 28252528 PMC5382495

[93] Hsue PY Ribaudo HJ Deeks SG Bell T Ridker PM Fichtenbaum C. Safety and impact of low-dose methotrexate on endothelial function and inflammation in individuals with treated human immunodeficiency virus: AIDS clinical trials group study A5314. Clin Infect Dis. (2019) 68:1877–86. 10.1093/cid/ciy781 30219823 PMC6522677

[94] Jacobson JM Bosinger SE Kang M Belaunzaran-Zamudio P Matining RM Wilson CC. The effect of chloroquine on immune activation and interferon signatures associated with HIV-1. AIDS Res Hum Retroviruses. (2016) 32:636–47. 10.1089/aid.2015.0336 26935044 PMC4931767

[95] Murray SM Down CM Boulware DR Stauffer WM Cavert WP Schacker TW. Reduction of immune activation with chloroquine therapy during chronic HIV infection. J Virol. (2010) 84:12082–6. 10.1128/JVI.01466-10 20844049 PMC2977889

[96] O'Brien MP Hunt PW Kitch DW Klingman K Stein JH Funderburg NT. A randomized placebo controlled trial of aspirin effects on immune activation in chronically human immunodeficiency virus-infected adults on virologically suppressive antiretroviral therapy. Open Forum Infect Dis. (2017) 4:ofw278. 10.1093/ofid/ofw278 28480270 PMC5414028

[97] Utay NS Kitch DW Yeh E Fichtenbaum CJ Lederman MM Estes JD. Telmisartan therapy does not improve lymph node or adipose tissue fibrosis more than continued antiretroviral therapy alone. J Infect Dis. (2018) 217:1770–81. 10.1093/infdis/jiy064 29401318 PMC5946950

[98] Capuron L Geisler S Kurz K Leblhuber F Sperner-Unterweger B Fuchs D. Activated immune system and inflammation in healthy ageing: relevance for tryptophan and neopterin metabolism. Curr Pharm Des. (2014) 20:6048–57. 10.2174/1381612820666140317110217 24641220

[99] Spencer ME Jain A Matteini A Beamer BA Wang NY Leng SX. Serum levels of the immune activation marker neopterin change with age and gender and are modified by race, BMI, and percentage of body fat. J Gerontol A Biol Sci Med Sci. (2010) 65:858–65. 10.1093/gerona/glq066 20478905 PMC2903784

