# Supplementary figures and images for: Associations Between Plasma Immunomodulatory and Inflammatory Mediators With VACS Index Scores Among Older HIV-Infected Adults on Antiretroviral Therapy
Source: Front Immunol. 2020 Jun 30;11:1321. doi: 10.3389/fimmu.2020.01321 (PMC7338430; doi:10.3389/fimmu.2020.01321)

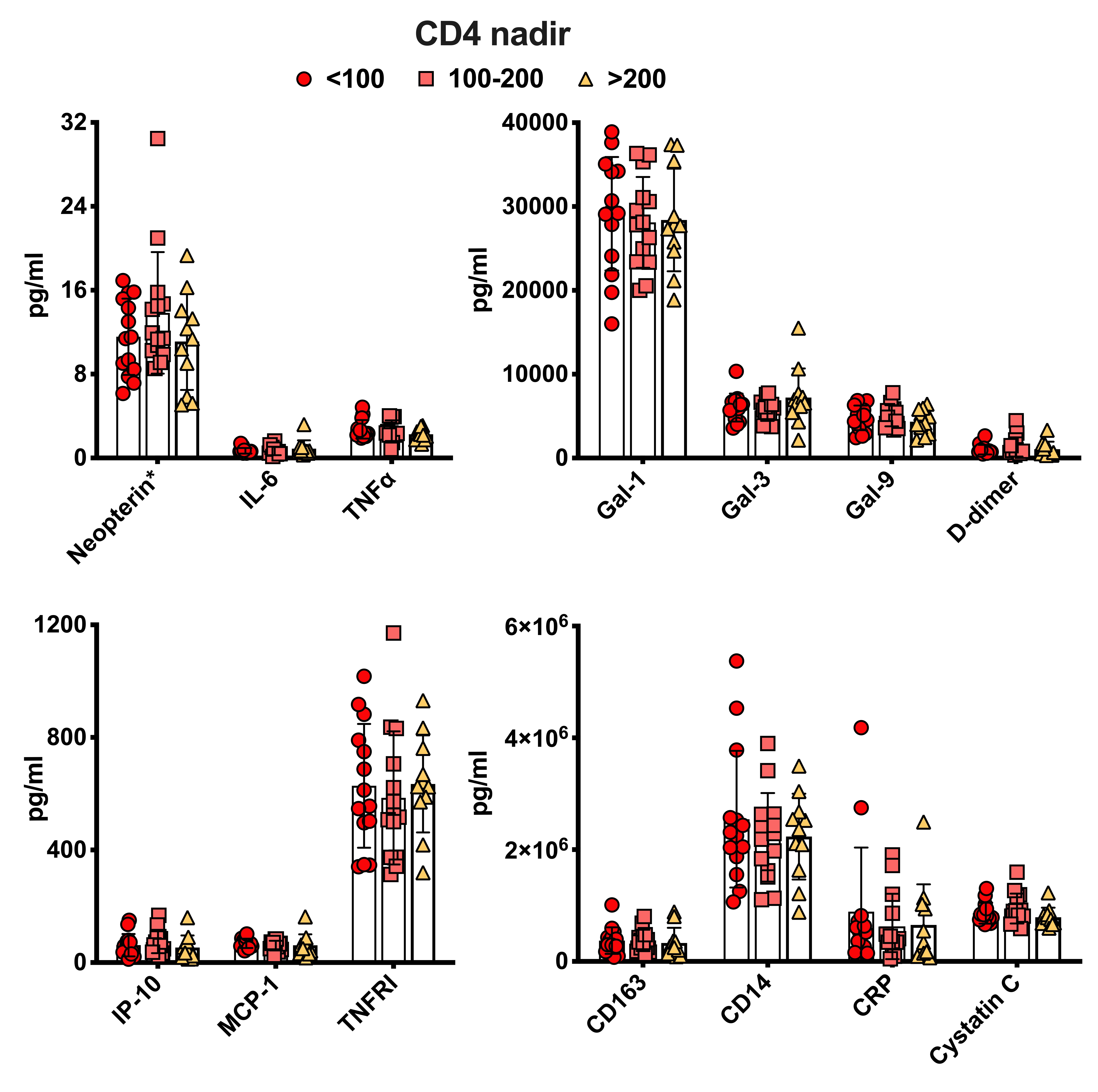

Supplement: Supplementary Figure 1 — Differences in soluble mediator levels in individuals according to CD4 nadir count. Associations among groups were analyzed via Kruskal-Wallis with Dunn's multiple comparison test. Neopterin concentration is in nMol/L. [file Image_1.JPEG]
